# Case Report: Congenital disseminated tuberculosis neonate born to tuberculosis- COVID-19 mother

**DOI:** 10.3389/fped.2022.941570

**Published:** 2022-10-28

**Authors:** Nelly Amalia Risan, Rilda Dwi Febrianda, Heda Melinda Nataprawira

**Affiliations:** Department of Child Health, Faculty of Medicine Universitas Padjadjaran Dr. Hasan Sadikin General Hospital, Bandung, West Java, Indonesia

**Keywords:** tuberculosis, COVID-19, meningitis, disseminated, congenital

## Abstract

We report a case of a 26-day-old male neonate with high suggestive of congenital disseminated tuberculosis (TB) [tuberculous meningitis (TBM), pulmonary TB, and ocular TB] at term, low birth weight born cesarean section from a confirmed COVID-19 and pulmonary TB mother who hospitalized in the isolation room and never seen her son for three weeks. The baby had a fever for two weeks after birth and a history of seizures. A nasopharyngeal swab for RT-PCR SARS-CoV-2 yielded a negative result. He was initially diagnosed as having a sepsis-like syndrome and then hospitalized. Chest x-ray revealed bilateral infiltrate, cerebrospinal fluid analysis (CSF) showed clear, cell count was 9 with dominant mononuclear cell, and gastric lavages did not yield acid-fast bacilli. X-pert MTB/RIF from gastric lavage specimen detected *Mycobacterium tuberculosis* rifampicin sensitive. Anti-tuberculosis drugs for TBM were started. Abdominal sonography identified multiple hypoechoic nodules in the liver and spleen. Neuroimaging studies did not identify hydrocephalus, meningeal enhancement, infarct, or tuberculoma. A Video-EEG examination showed electrical seizure after initiation of phenobarbital. Video-EEG evaluation showed no epileptiform discharge. Upon follow-up, he showed slightly delayed motor development, pan-uveitis, retinal detachment, and cataracts. We assumed that ocular TB resulted from a paradoxical reaction following TB treatment. Retinal detachment was improved and lens replacement was done.

## Introduction

The COVID-19 pandemic has had a major impact on healthcare delivery worldwide. The risks to the fetus due to COVID-19 during pregnancy are not fully known, and co-infection with pulmonary tuberculosis can further complicate the situation (1). The actual incidence of congenital TB has rarely been reported (2). It has a significant mortality rate of up to 50% (3, 4). *Mycobacterium tuberculosis* can spread beyond the pulmonary into the systemic circulation, causing the dissemination of infection involving the gastrointestinal tract, spleen, kidney, adrenals, bone marrow, and meninges (3, 5). The sepsis-like syndrome can be present if there is widespread dissemination (5). Disseminated TB is an important cause of morbidity and mortality in developing countries, especially among children under five years (5). Early identification and management are essential to prevent ongoing disease transmission (5). The diagnosis of congenital TB is challenging due to the non-specific presentation in infants, hence it is necessary to monitor pregnant women with TB during this pandemic with early detection of TB in newborns to prevent further and more severe TB (2–4, 6).

During the pandemic, TB and COVID-19 tests can be carried out simultaneously because of the same clinical features of both diseases, simultaneous exposure to both diseases, and the presence of risk factors for a poor outcome for both diseases (7).

## Case presentation

A 26-day-old male neonate was brought with complaints of recurrent fever and a history of seizures since two weeks old. He was born at term, 2,400 gr, *via* cesarean section to a confirmed COVID-19 and TB mother. A nasopharyngeal swab for RT-PCR SARS CoV-2 of the baby was negative. He was discharged in good condition from the hospital on the 3rd day and was separated from his mother for 3 weeks. He was never given TB prophylaxis after birth.

**Figure 1:**
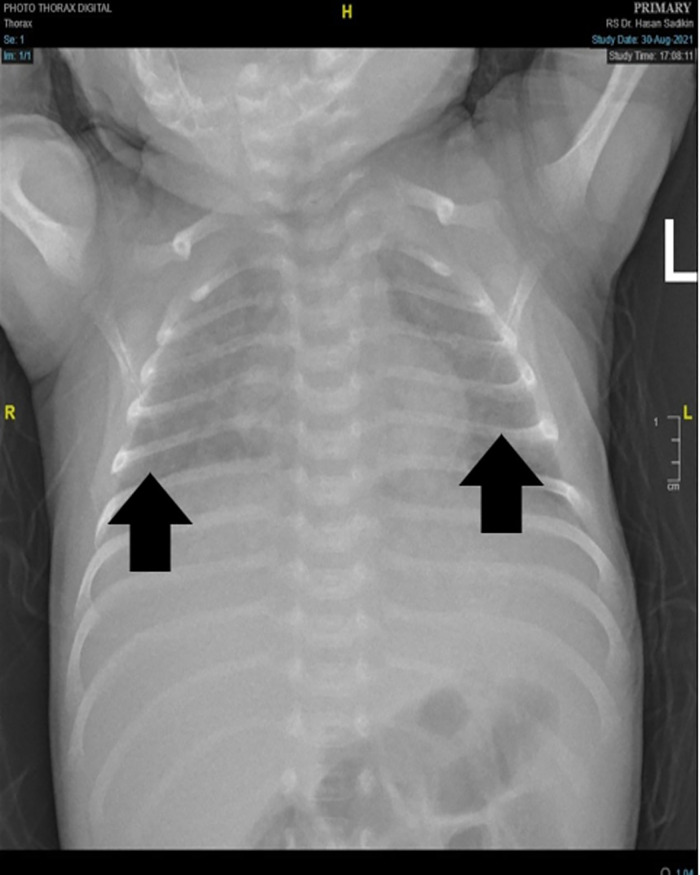
Chest x-Ray. showed inhomogeneous opaque coverings within upper and lower lung fields bilaterally (black arrow).

**Figure 2:**
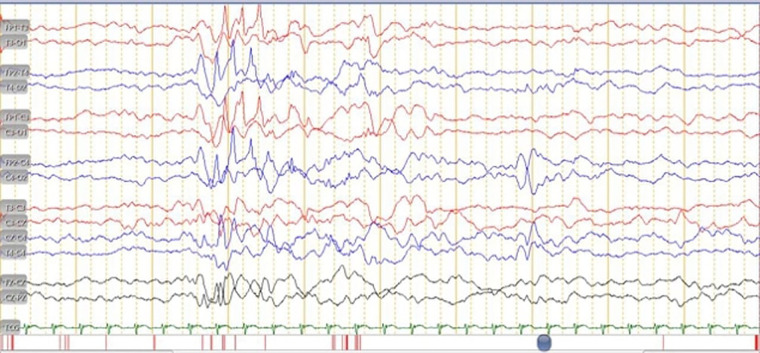
EEG in a month-old male with congenital disseminated TB showed there was an electrical seizure.

At the presentation, he was alert and feverish. No abnormal respiratory findings nor seizures, but tachycardia was identified. An abdominal examination revealed liver and spleen enlargement. The laboratory findings showed decreased platelet counts, an elevated liver function test, and an increase in total bilirubin and c-reactive protein. The HIV serology result was non-reactive, while the viral load was not investigated. A chest x-ray revealed bilateral pneumonia (Figure 1) and an abdominal radiograph concluded a liver enlargement. He was initially diagnosed with late-onset neonatal sepsis and was given a course of empiric antibiotics. The patient had previously received phenobarbital 20 mg/kg body weight at the previous hospital and was immediately referred to our hospital. The drug was discontinued when the seizures stopped without an EEG examination. On the 3rd day of hospitalization, he had tonic posturing, suggestive of meningitis. We did a lumbar puncture and the CSF was clear with increased cell count (9 cells/HPF, mononuclear cells dominant). An X-pert MTB/RIF from gastric lavage detected rifampicin-sensitive *Mycobacterium tuberculosis*. He has been diagnosed with TBM and decided to start a TB drug regimen. However, we could not give rifampicin and isoniazid due to the increase in ALT and direct bilirubin level, so he was given levofloxacin and ethambutol. The patient did not show any sign of a seizure. However, the video-EEG result showed an electrical seizure (Figure 2). A brain MRI did not show any abnormalities or signs of meningitis.

After starting the TB regimen, his condition slowly improved. The fever was down and there were no clinical seizures. Abdominal sonography showed multiple hypoechoic nodules that were suggestive of liver and splenic TB. He was then diagnosed with suspected congenital disseminated TB. During hospitalization, his liver function improved. Rifampicin and isoniazid were introduced and were well tolerated. After 30 days of hospitalization, evaluation using the EEG showed no epileptiform discharge, and phenobarbital was discontinued. On day 33, he was discharged in good condition.

**Figure 3:**
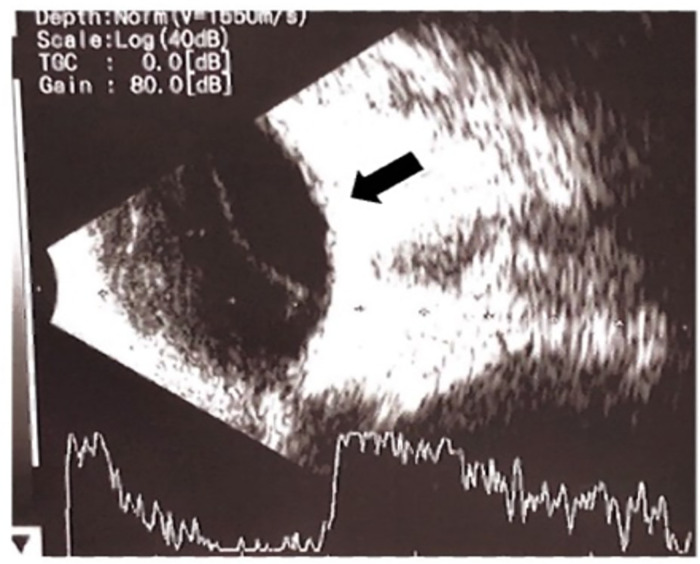
Ultrasound examination of the left eye showed pan-uveitis developed exudative retinal detachment.

At three months old, he showed poor neurodevelopmental progress with gross motor delay and poor visual acuity test results (i.e., not following light). We referred the patient for a complete ophthalmology examination which revealed pan-uveitis complicated with cataracts, and exudative retinal detachment of the left eye (Figure 3). He was given levofloxacin, prednisolone acetate, and homatropine hydrobromide eye drops. He also underwent lens replacements for cataracts and the retinal detachment was resolved.

At six months, a follow-up MRI was normal. Brain Evoked Response Audiometry (BERA) was also normal. Amiel-Tison's neurological assessment showed mild abnormal motoric development with slight spasticity. He still takes the TB regimen regularly and we continue the therapy for 12 months.

His mother had a cough symptom since the 6th month of pregnancy but was not evaluated for TB. A cesarean section was planned because of breech presentation. Three days before the cesarean section, she had a low-grade fever, and the PCR result prior to the cesarean delivery was positive. Because the mother's chest x-ray after the delivery showed an infiltrate that was suggestive of TB, further evaluation for TB was done. The AFB sputum smear result was positive, so she was started on a TB regimen. Unfortunately, this was not accompanied by a TB investigation for the baby. The baby was discharged without TB prophylaxis. The placenta was not examined in the previous hospital. A further contact TB investigation result from her family was negative.

## Discussion

Pregnant women are at increased risk of developing TB, and this is associated with poor outcomes including premature birth, intrauterine growth retardation, and low birth weight. COVID-19 and tuberculosis co-infection in pregnancy is rare (1, 8). In the general population, one study from China reveals the TB prevalence among COVID-19 patients ranged between 0.47 to 4.47% %. TB status might play a role in the development of COVID-19 infection, exacerbation of the course of the disease, and vice-versa (9).

Here, we described the outcome of an infant with congenital disseminated TB born from a mother with COVID-19 and TB co-infection. Immune suppression caused by the SARS-CoV-2 virus may result in certain difficulties in the diagnosis and treatment of tuberculosis. Furthermore, long-term lymphopenia, hyperinflammation, lung tissue injury, and imbalance in CD4+ T cell subsets associated with COVID-19 could propagate M. tuberculosis infection and disease progression (10). The nasopharyngeal swab for RT-PCR SARS CoV-2 of the baby was negative, which likely indicated that no vertical transmission occurred (11). Congenital TB is a fatal disease that must be diagnosed as early as possible to avoid devastating outcomes (2–4). In a newborn child, the clinical presentation of the disease is non-specific, which often delays the diagnosis (6, 12). The transmission is most commonly from hematogenous spread *via* the umbilical vein, infected amniotic fluid aspiration, or ingestion of infected secretions (6, 12). The primary complex in the liver, along with caseating granuloma, is the definitive lesion of congenital tuberculosis (2–4). Unfortunately, we cannot prove this case was congenital in origin as we did not perform the liver biopsy and placental histopathology examination to find caseating tubercles for diagnosing congenital TB. The suspicion of TB in pregnant mothers is necessary for diagnosing TB in neonates and must be considered in countries where TB incidence is high (13). The most common symptoms attributed to congenital TB are fever (70%), hepatic and/or splenic enlargement (67%), and lethargy with periods of irritability (40%), as identified in our case (3, 5).

Cantwell modified the criteria diagnosis of congenital TB; it requires (a) *Mycobacterium tuberculosis* lesion and (b) 1 of the following secondary findings: (1) Primary hepatic complex (caseating granuloma) on biopsy, (2) lesions from any source (i.e., pulmonary, hepatic, and skin) in the first weeks after birth, (3) exclusion of postnatal transmission through a thorough investigation of contacts, or (4) TB infection of the maternal genital tract and/or placenta (3, 14). Congenital TB, in this case, has not been established because the histopathological examination of the placenta was not performed. However, we excluded post-natal transmission by thorough investigation through history taking and AFB sputum smear. In this case, we assumed he had TB congenitally transmitted, and the patient had developed symptoms of TB since the age of 2 weeks before contact with his mother. The age at the beginning of congenital TB is not uniform. Infants with congenital TB may be asymptomatic at birth, but symptoms can occur within days to weeks after birth. This is due to the different immune statuses of each newborn and the onset of disease may be slower in some children (15). The symptoms of congenital TB mainly occur until 3 months after birth, at an average age of 28 days with the longest duration between birth and the onset of symptoms being 154 days (15). He had severe manifestations of congenital disseminated tuberculosis (i.e., TBM, pulmonary TB, and ocular TB).

Active TB in pregnancy is associated with adverse maternal and fetal outcomes. Early diagnosis of TB is important to prevent significant maternal and perinatal complications. Newborns from mothers with pulmonary TB must be given tuberculosis preventive treatment (TPT) and should be separated until both have been evaluated (14, 16).. Unfortunately, the patient did not receive TPT from the previous hospital.

Congenital TB could develop into meningitis TB with seizures as its primary symptoms; therefore we should consider the possibility of meningitis TB (12, 14). Neonatal seizures, if prolonged and untreated, can cause permanent damage due to decreased oxygen flow and excessive brain cell activity. Subtle or electrical seizures are common in neonates, described as subtle because the symptoms are frequently overlooked (17). Electrographic or electrical seizures, namely EEG electrical seizure activity without apparent clinical manifestation, are more common after the initiation of an antiepileptic drug such as phenobarbital (17).

The anti-epileptic drug can suppress the clinical manifestation of seizure but not the EEG ictal discharge (17). An electrographic seizure could be a sign of poor prognosis (17). We suspected that ocular TB in this patient was due to a paradoxical reaction following TB treatment. In the beginning, the patient's eyes were found to be normal but during monitoring, there were complications in the patient's eyes (i.e., pan-uveitis, cataracts, and retinal detachment). A paradoxical reaction in patients with TBM is characterized by the worsening of pre-existing tuberculous lesions or the appearance of new tuberculous lesions in patients whose clinical symptoms initially improved and had been on anti-tuberculosis treatment for at least 10 days (18). This case illustrates the important obstacles in managing a newborn with TB. A placenta examination was not performed, resulting in the delay of diagnosis, and not giving TPT may worsen the patient's outcome. A bone marrow puncture (BMP) was not performed due to decreasing platelet count of <50.000/mm^3^. This case showed up on monitoring, and various complications were discovered. Therefore, this is an important issue that must be considered to improve the diagnosis and treatment that can reduce morbidity and mortality in TBM.

Our limitation is that we do not know whether TB in this patient is of congenital origin or not because there is no confirmation data from placental histopathology.

## Conclusions

In this case, we described a baby from a COVID-19 and TB-positive mother with severe congenital TB. It is necessary to monitor pregnant women with TB during this pandemic and early detection of TB in newborns to prevent further and more severe TB. It is important to follow up with TB patients to ensure that a paradoxical reaction does not occur as it did with this patient. Despite the severity of congenital TB, prompt and rigorous treatment of seizures and the management of eye complications had good outcomes in this case.

## Data Availability

The original contributions presented in the study are included in the article/Supplementary Material, further inquiries can be directed to the corresponding author/s.
